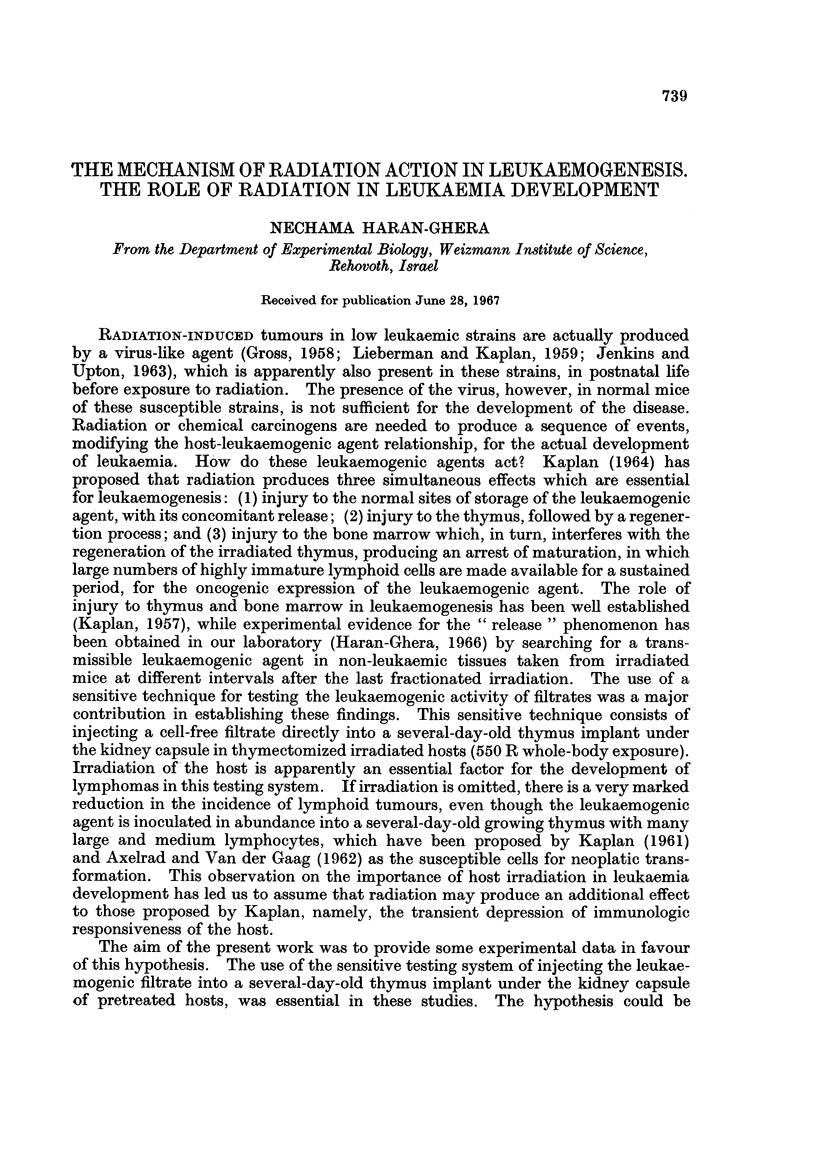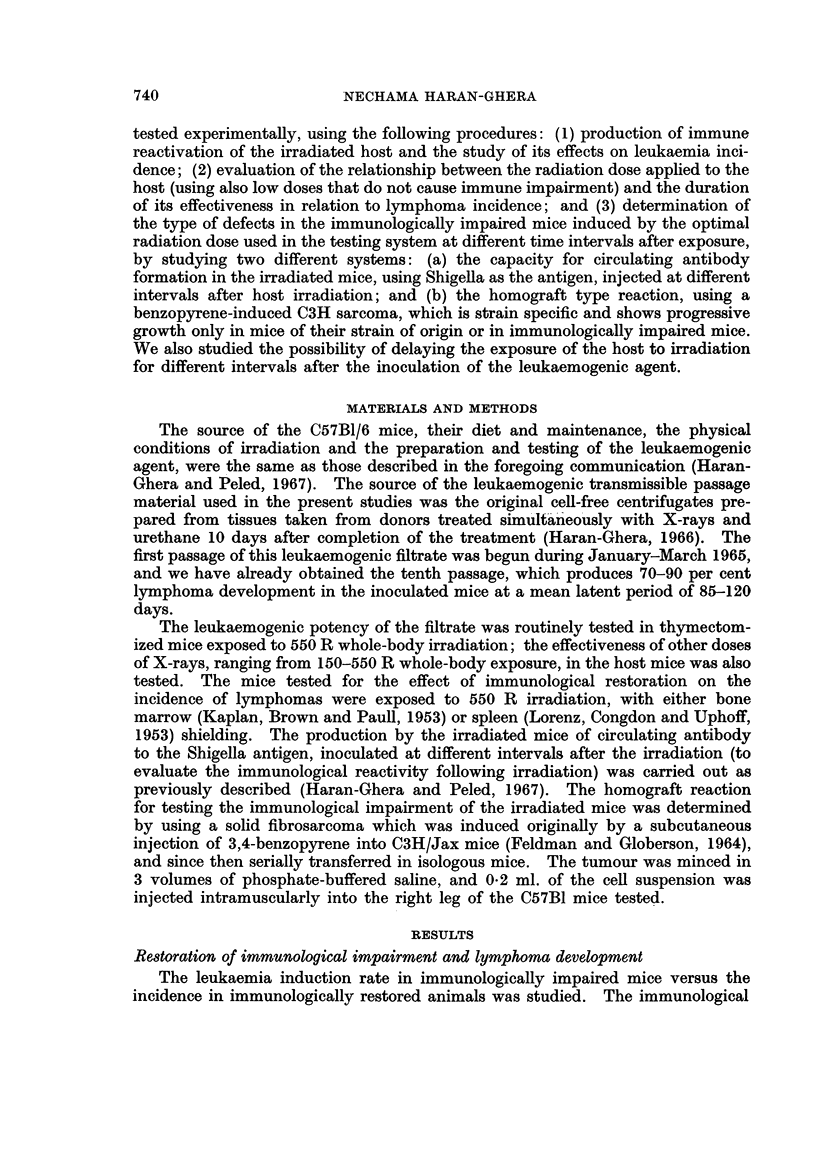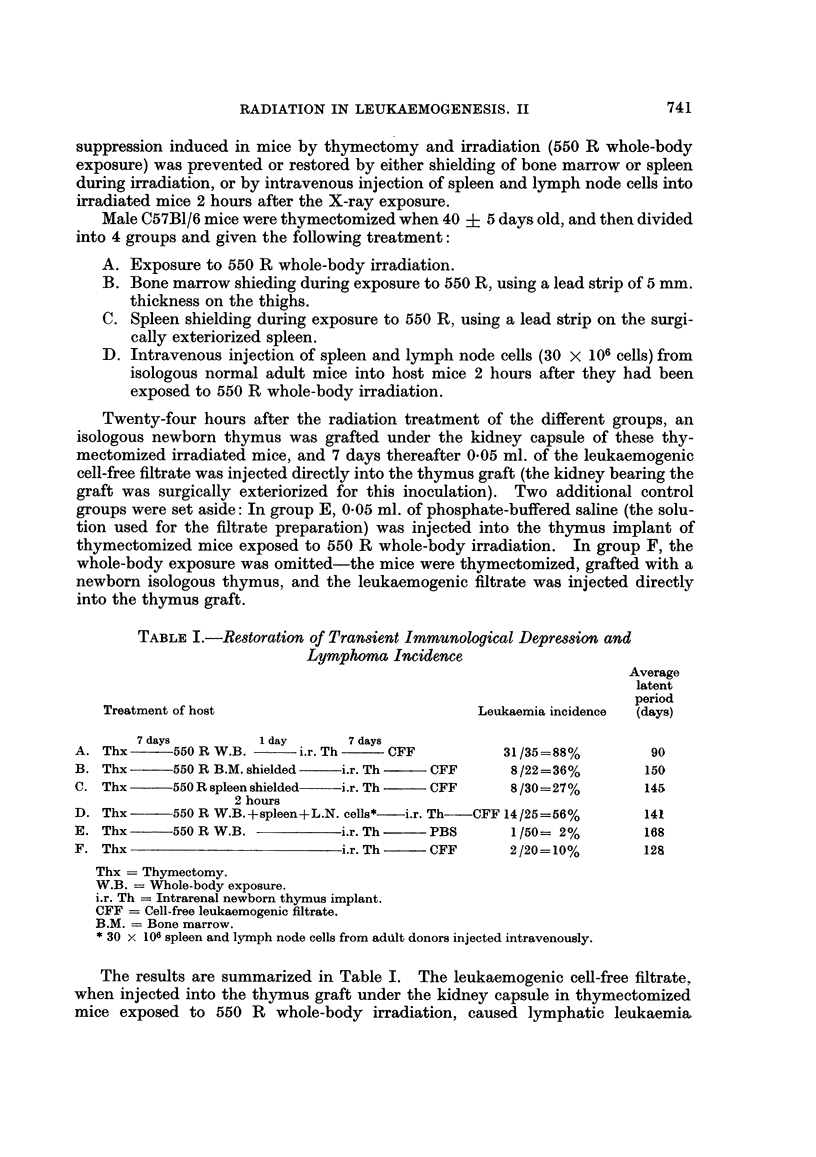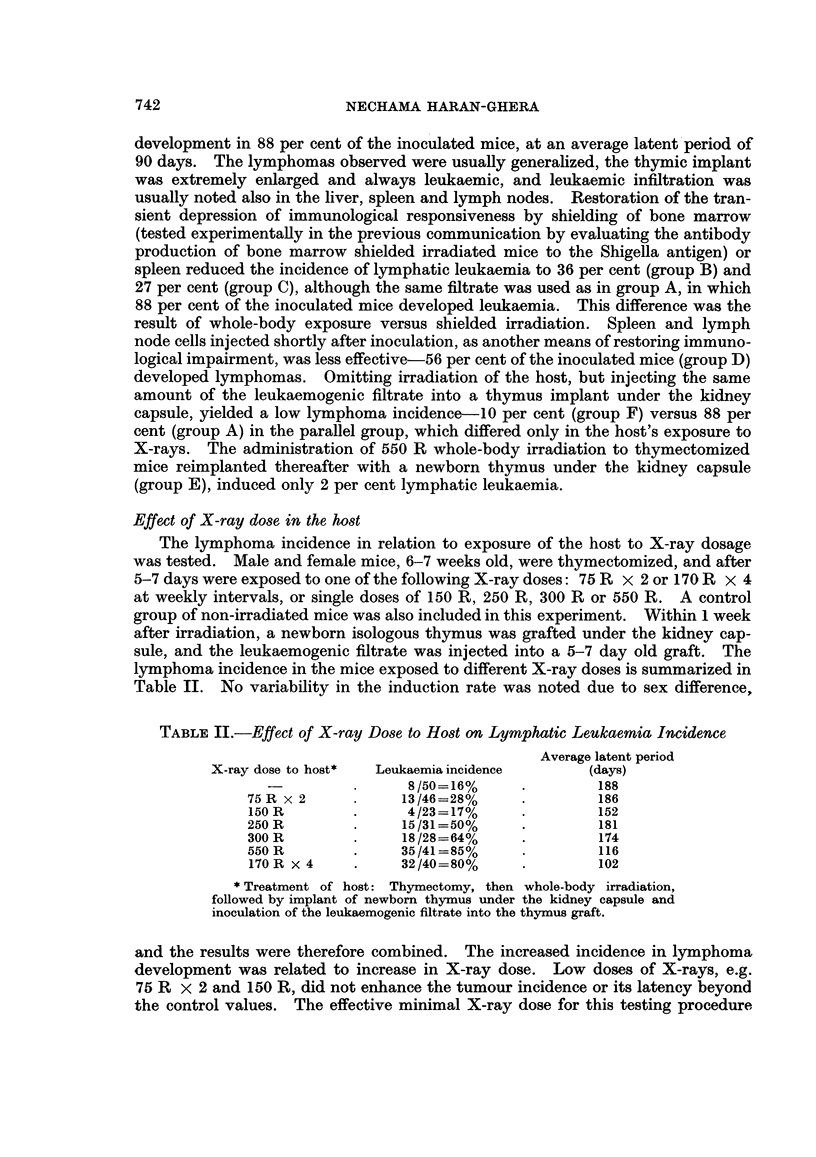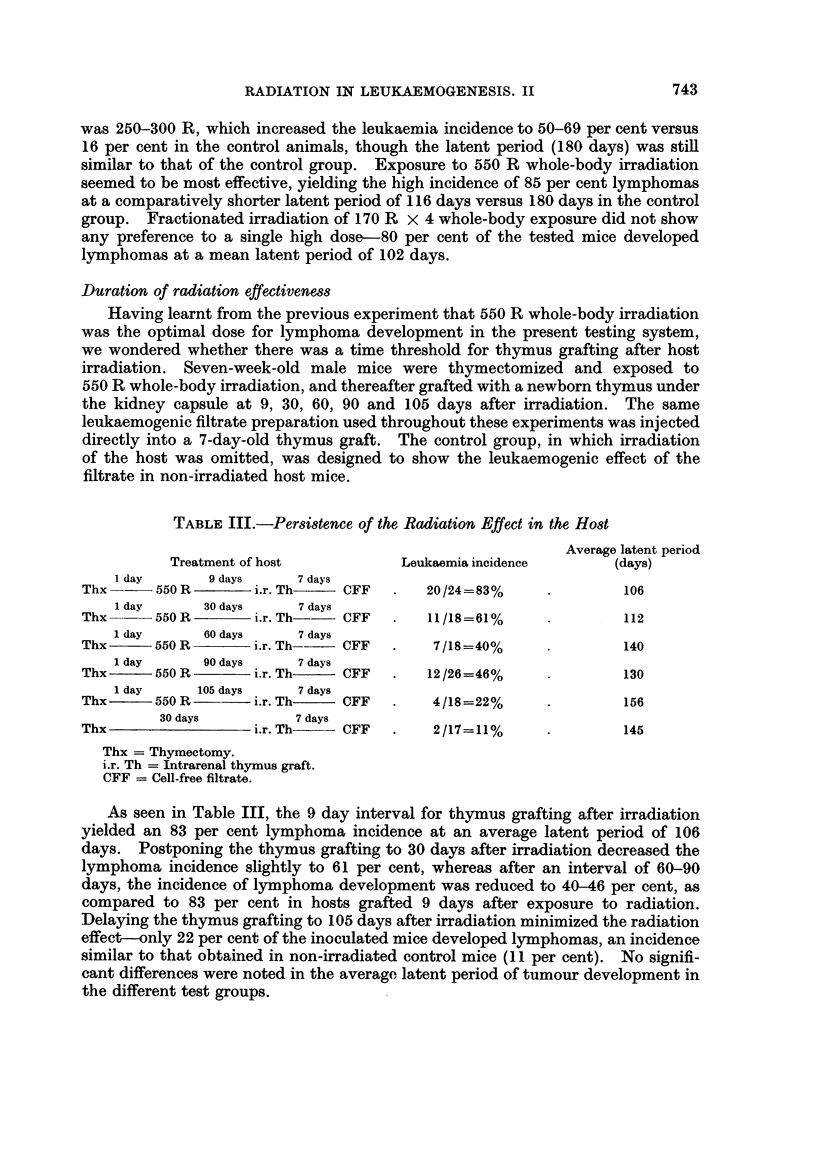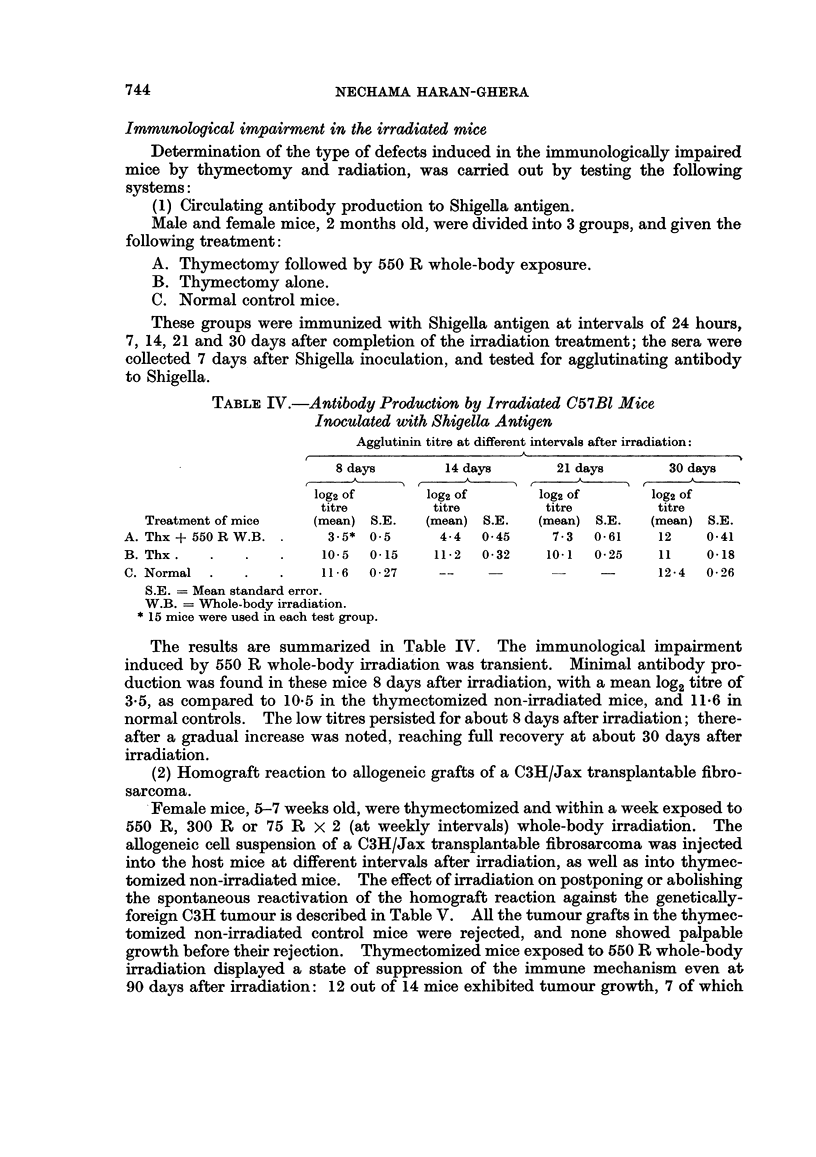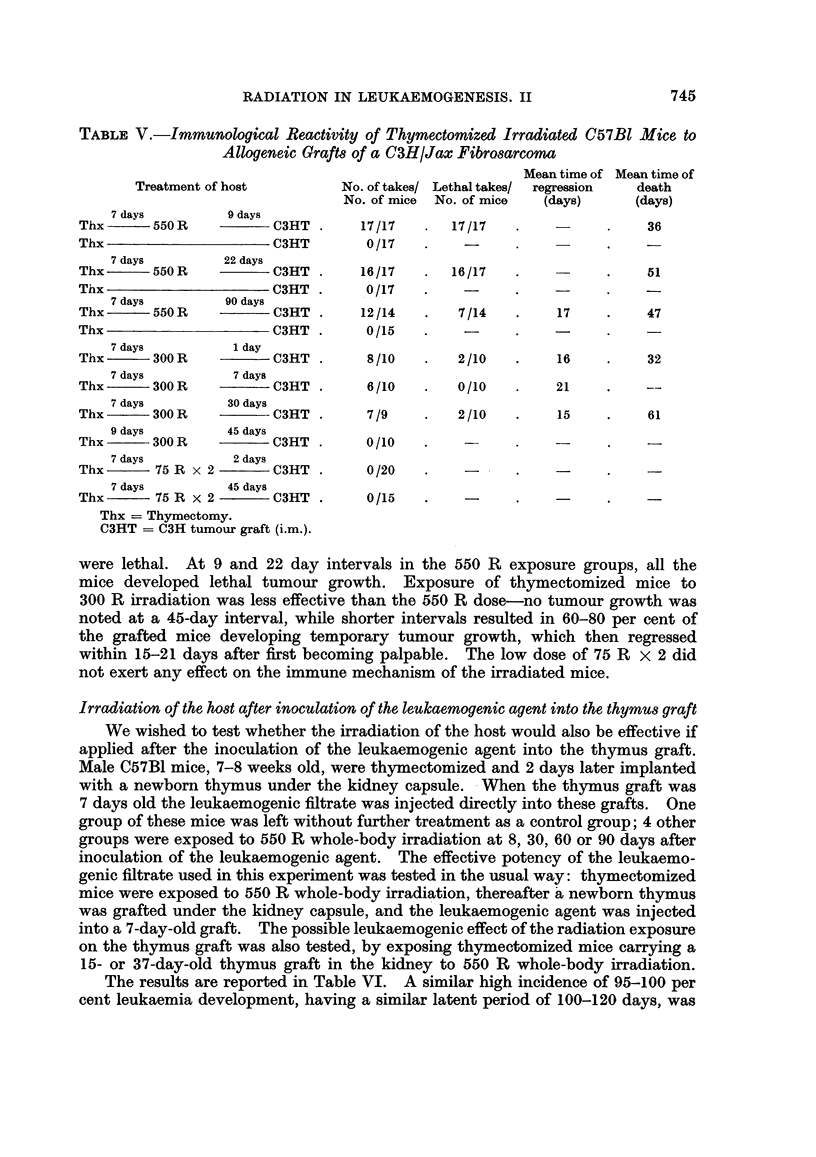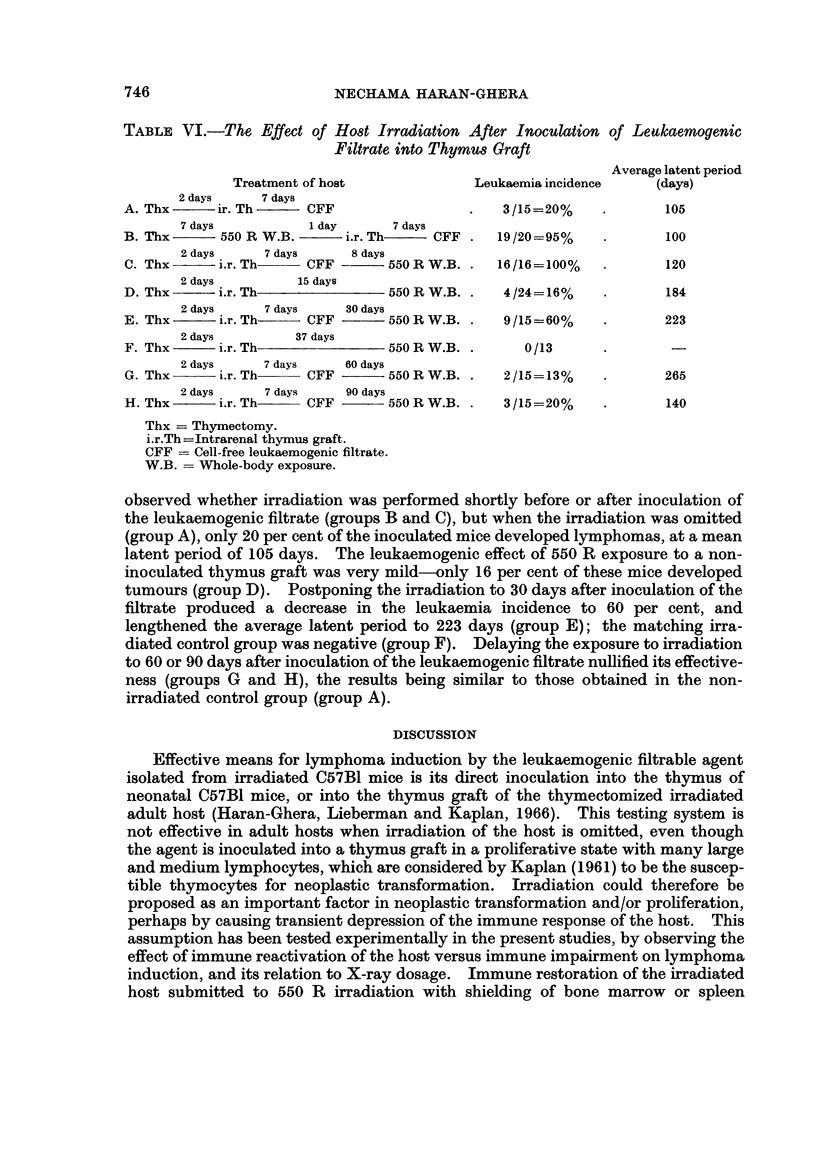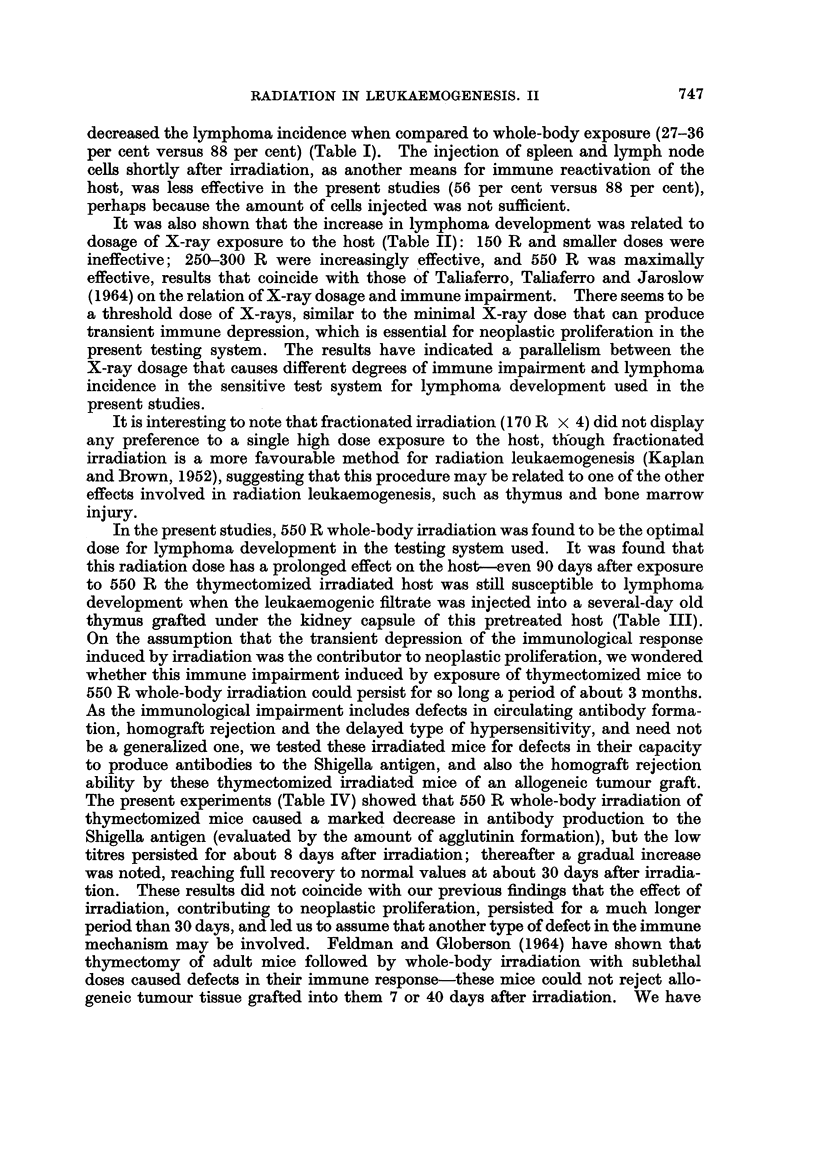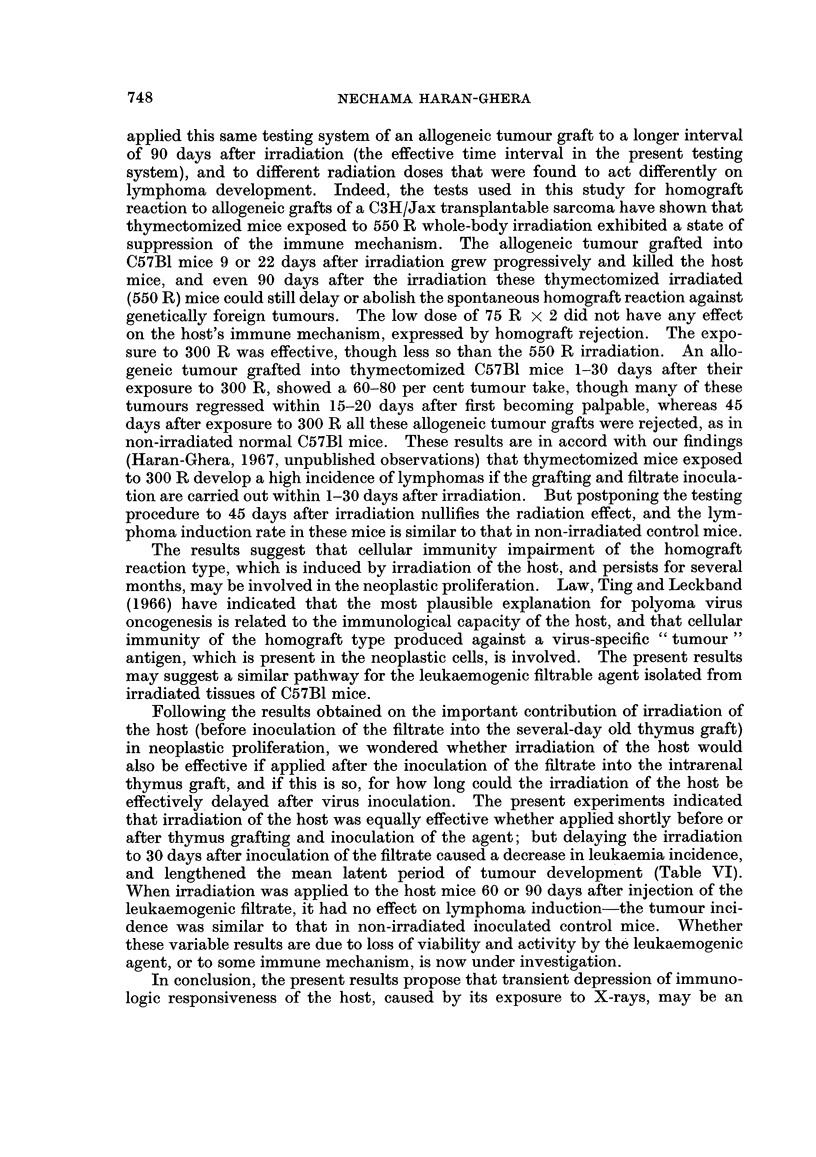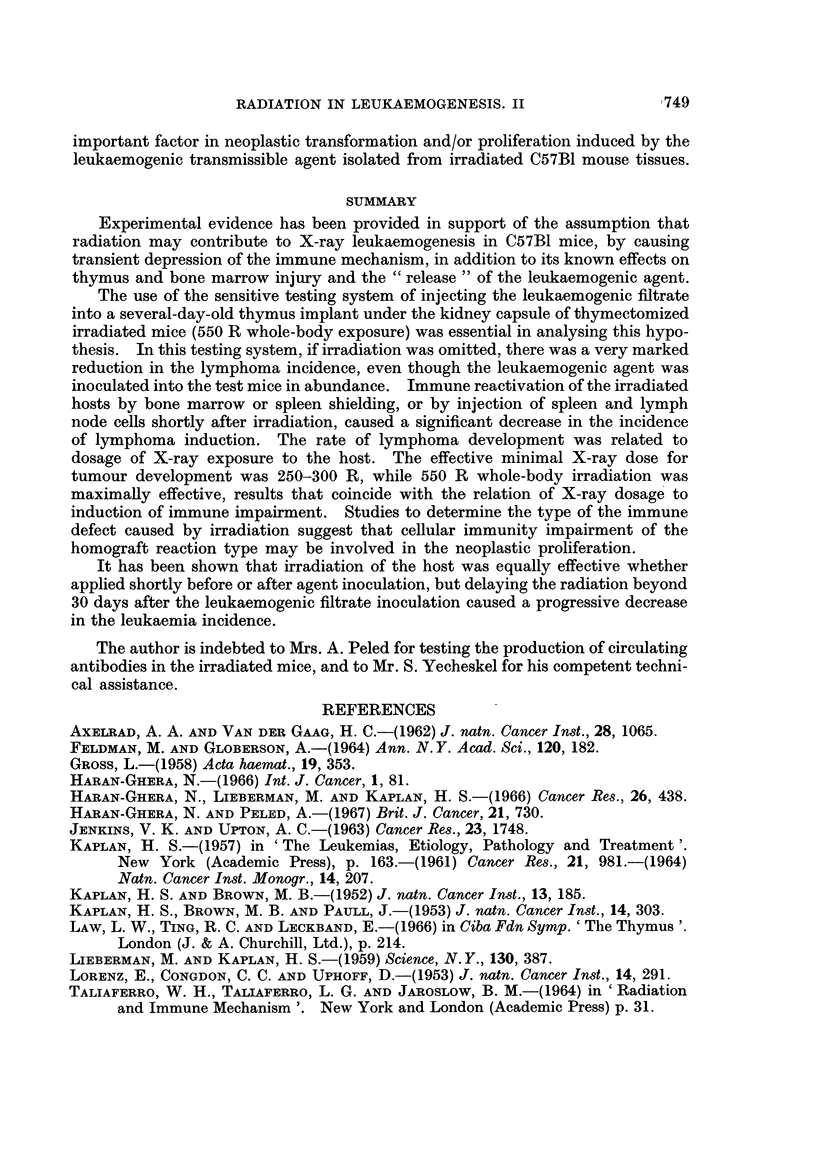# The mechanism of radiation action in leukaemogenesis. The role of radiation in leukaemia development.

**DOI:** 10.1038/bjc.1967.86

**Published:** 1967-12

**Authors:** N. Haran-Ghera


### THE MECHANISM OF RADIATION ACTION IN LEUKAEMOGENESIS. THE ROLE OF RADIATION IN LEUKAEMIA DEVELOPMENT

NECHAMA HARAN-GHERA

*From the Department of Experimental Biology, Weizmann Institute of Science, Rehovoth, Israel*

Received for publication June 28, 1967

RADIATION-INDUCED tumours in low leukaemic strains are actually produced by a virus-like agent (Gross, 1958; Lieberman and Kaplan, 1959; Jenkins and Upton, 1963), which is apparently also present in these strains, in postnatal life before exposure to radiation. The presence of the virus, however, in normal mice of these susceptible strains, is not sufficient for the development of the disease. Radiation or chemical carcinogens are needed to produce a sequence of events, modifying the host-leukaemogenic agent relationship, for the actual development of leukaemia. How do these leukaemogenic agents act? Kaplan (1964) has proposed that radiation produces three simultaneous effects which are essential for leukaemogenesis: (1) injury to the normal sites of storage of the leukaemogenic agent, with its concomitant release; (2) injury to the thymus, followed by a regenertion process; and (3) injury to the bone marrow which, in turn, interferes with the regeneration of the irradiated thymus, producing an arrest of maturation, in which large numbers of highly immature lymphoid cells are made available for a sustained period, for the oncogenic expression of the leukaemogenic agent. The role of injury to thymus and bone marrow in leukaemogenesis has been well established (Kaplan, 1957), while experimental evidence for the " release " phenomenon has been obtained in our laboratory (Haran-Ghera, 1966) by searching for a transmissible leukaemogenic agent in non-leukaemic tissues taken from irradiated mice at different intervals after the last fractionated irradiation. The use of a sensitive technique for testing the leukaemogenic activity of filtrates was a major contribution in establishing these findings. This sensitive technique consists of injecting a cell-free filtrate directly into a several-day-old thymus implant under the kidney capsule in thymectomized irradiated hosts (550 R whole-body exposure). Irradiation of the host is apparently an essential factor for the development of lymphomas in this testing system. If irradiation is omitted, there is a very marked reduction in the incidence of lymphoid tumours, even though the leukaemogenic agent is inoculated in abundance into a several-day-old growing thymus with many large and medium lymphocytes, which have been proposed by Kaplan (1961) and Axelrad and Van der Gaag (1962) as the susceptible cells for neoplatic transformation. This observation on the importance of host irradiation in leukaemia development has led us to assume that radiation may produce an additional effect to those proposed by Kaplan, namely, the transient depression of immunologic responsiveness of the host.

The aim of the present work was to provide some experimental data in favour of this hypothesis. The use of the sensitive testing system of injecting the leukaemogenic filtrate into a several-day-old thymus implant under the kidney capsule of pretreated hosts, was essential in these studies. The hypothesis could be tested experimentally, using the following procedures: (1) production of immune reactivation of the irradiated host and the study of its effects on leukaemia incidence; (2) evaluation of the relationship between the radiation dose applied to the host (using also low doses that do not cause immune impairment) and the duration of its effectiveness in relation to lymphoma incidence; and (3) determination of the type of defects in the immunologically impaired mice induced by the optimal radiation dose used in the testing system at different time intervals after exposure, by studying two different systems: (a) the capacity for circulating antibody formation in the irradiated mice, using Shigella as the antigen, injected at different intervals after host irradiation; and (b) the homograft type reaction, using a benzopyrene-induced C3H sarcoma, which is strain specific and shows progressive growth only in mice of their strain of origin or in immunologically impaired mice. We also studied the possibility of delaying the exposure of the host to irradiation for different intervals after the inoculation of the leukaemogenic agent.

### MATERIALS AND METHODS

The source of the C57Bl/6 mice, their diet and maintenance, the physical conditions of irradiation and the preparation and testing of the leukaemogenic agent, were the same as those described in the foregoing communication (Haran-Ghera and Peled, 1967). The source of the leukaemogenic transmissible passage material used in the present studies was the original cell-free centrifugates prepared from tissues taken from donors treated simultaneously with X-rays and urethane 10 days after completion of the treatment (Haran-Ghera, 1966). The first passage of this leukaemogenic filtrate was begun during January–March 1965, and we have already obtained the tenth passage, which produces 70–90 per cent lymphoma development in the inoculated mice at a mean latent period of 85–120 days.

The leukaemogenic potency of the filtrate was routinely tested in thymectomized mice exposed to 550 R whole-body irradiation; the effectiveness of other doses of X-rays, ranging from 150–550 R whole-body exposure, in the host mice was also tested. The mice tested for the effect of immunological restoration on the incidence of lymphomas were exposed to 550 R irradiation, with either bone marrow (Kaplan, Brown and Paull, 1953) or spleen (Lorenz, Congdon and Uphoff, 1953) shielding. The production by the irradiated mice of circulating antibody to the Shigella antigen, inoculated at different intervals after the irradiation (to evaluate the immunological reactivity following irradiation) was carried out as previously described (Haran-Ghera and Peled, 1967). The homograft reaction for testing the immunological impairment of the irradiated mice was determined by using a solid fibrosarcoma which was induced originally by a subcutaneous injection of 3,4-benzopyrene into C3H/Jax mice (Feldman and Globerson, 1964), and since then serially transferred in isologous mice. The tumour was minced in 3 volumes of phosphate-buffered saline, and 0·2 ml. of the cell suspension was injected intramuscularly into the right leg of the C57Bl mice tested.

### RESULTS

### *Restoration of immunological impairment and lymphoma development*

The leukaemia induction rate in immunologically impaired mice versus the incidence in immunologically restored animals was studied. The immunological suppression induced in mice by thymectomy and irradiation (550 R whole-body exposure) was prevented or restored by either shielding of bone marrow or spleen during irradiation, or by intravenous injection of spleen and lymph node cells into irradiated mice 2 hours after the X-ray exposure.

Male C57Bl/6 mice were thymectomized when 40 $\pm$ 5 days old, and then divided into 4 groups and given the following treatment:

A. Exposure to 550 R whole-body irradiation.
B. Bone marrow shieding during exposure to 550 R, using a lead strip of 5 mm. thickness on the thighs.
C. Spleen shielding during exposure to 550 R, using a lead strip on the surgically exteriorized spleen.
D. Intravenous injection of spleen and lymph node cells (30 $\times$ $10^6$ cells) from isologous normal adult mice into host mice 2 hours after they had been exposed to 550 R whole-body irradiation.

Twenty-four hours after the radiation treatment of the different groups, an isologous newborn thymus was grafted under the kidney capsule of these thymectomized irradiated mice, and 7 days thereafter 0·05 ml. of the leukaemogenic cell-free filtrate was injected directly into the thymus graft (the kidney bearing the graft was surgically exteriorized for this inoculation). Two additional control groups were set aside: In group E, 0·05 ml. of phosphate-buffered saline (the solution used for the filtrate preparation) was injected into the thymus implant of thymectomized mice exposed to 550 R whole-body irradiation. In group F, the whole-body exposure was omitted—the mice were thymectomized, grafted with a newborn isologous thymus, and the leukaemogenic filtrate was injected directly into the thymus graft.

TABLE I.—*Restoration of Transient Immunological Depression and Lymphoma Incidence*

| | Treatment of host | Leukaemia incidence | Average latent period (days) |
|---|---|---|---|
| A. | Thx —7 days— 550 R W.B. —1 day— i.r. Th —7 days— CFF | 31/35=88% | 90 |
| B. | Thx —— 550 R B.M. shielded —— i.r. Th —— CFF | 8/22=36% | 150 |
| C. | Thx —— 550 R spleen shielded —— i.r. Th —— CFF | 8/30=27% | 145 |
| D. | Thx —— 550 R W.B. —2 hours— +spleen+L.N. cells* —— i.r. Th —— CFF | 14/25=56% | 141 |
| E. | Thx —— 550 R W.B. —— i.r. Th —— PBS | 1/50= 2% | 168 |
| F. | Thx —— i.r. Th —— CFF | 2/20=10% | 128 |

Thx = Thymectomy.
W.B. = Whole-body exposure.
i.r. Th = Intrarenal newborn thymus implant.
CFF = Cell-free leukaemogenic filtrate.
B.M. = Bone marrow.
* 30 $\times$ $10^6$ spleen and lymph node cells from adült donors injected intravenously.

The results are summarized in Table I. The leukaemogenic cell-free filtrate, when injected into the thymus graft under the kidney capsule in thymectomized mice exposed to 550 R whole-body irradiation, caused lymphatic leukaemia development in 88 per cent of the inoculated mice, at an average latent period of 90 days. The lymphomas observed were usually generalized, the thymic implant was extremely enlarged and always leukaemic, and leukaemic infiltration was usually noted also in the liver, spleen and lymph nodes. Restoration of the transient depression of immunological responsiveness by shielding of bone marrow (tested experimentally in the previous communication by evaluating the antibody production of bone marrow shielded irradiated mice to the Shigella antigen) or spleen reduced the incidence of lymphatic leukaemia to 36 per cent (group B) and 27 per cent (group C), although the same filtrate was used as in group A, in which 88 per cent of the inoculated mice developed leukaemia. This difference was the result of whole-body exposure versus shielded irradiation. Spleen and lymph node cells injected shortly after inoculation, as another means of restoring immunological impairment, was less effective—56 per cent of the inoculated mice (group D) developed lymphomas. Omitting irradiation of the host, but injecting the same amount of the leukaemogenic filtrate into a thymus implant under the kidney capsule, yielded a low lymphoma incidence—10 per cent (group F) versus 88 per cent (group A) in the parallel group, which differed only in the host's exposure to X-rays. The administration of 550 R whole-body irradiation to thymectomized mice reimplanted thereafter with a newborn thymus under the kidney capsule (group E), induced only 2 per cent lymphatic leukaemia.

*Effect of X-ray dose in the host*

The lymphoma incidence in relation to exposure of the host to X-ray dosage was tested. Male and female mice, 6–7 weeks old, were thymectomized, and after 5–7 days were exposed to one of the following X-ray doses: 75 R × 2 or 170 R × 4 at weekly intervals, or single doses of 150 R, 250 R, 300 R or 550 R. A control group of non-irradiated mice was also included in this experiment. Within 1 week after irradiation, a newborn isologous thymus was grafted under the kidney capsule, and the leukaemogenic filtrate was injected into a 5–7 day old graft. The lymphoma incidence in the mice exposed to different X-ray doses is summarized in Table II. No variability in the induction rate was noted due to sex difference,

TABLE II.—*Effect of X-ray Dose to Host on Lymphatic Leukaemia Incidence*

| X-ray dose to host* | Leukaemia incidence | Average latent period (days) |
|---|---|---|
| — | 8/50=16% | 188 |
| 75 R × 2 | 13/46=28% | 186 |
| 150 R | 4/23=17% | 152 |
| 250 R | 15/31=50% | 181 |
| 300 R | 18/28=64% | 174 |
| 550 R | 35/41=85% | 116 |
| 170 R × 4 | 32/40=80% | 102 |

* Treatment of host: Thymectomy, then whole-body irradiation, followed by implant of newborn thymus under the kidney capsule and inoculation of the leukaemogenic filtrate into the thymus graft.

and the results were therefore combined. The increased incidence in lymphoma development was related to increase in X-ray dose. Low doses of X-rays, e.g. 75 R × 2 and 150 R, did not enhance the tumour incidence or its latency beyond the control values. The effective minimal X-ray dose for this testing procedure was 250–300 R, which increased the leukaemia incidence to 50–69 per cent versus 16 per cent in the control animals, though the latent period (180 days) was still similar to that of the control group. Exposure to 550 R whole-body irradiation seemed to be most effective, yielding the high incidence of 85 per cent lymphomas at a comparatively shorter latent period of 116 days versus 180 days in the control group. Fractionated irradiation of 170 R × 4 whole-body exposure did not show any preference to a single high dose—80 per cent of the tested mice developed lymphomas at a mean latent period of 102 days.

*Duration of radiation effectiveness*

Having learnt from the previous experiment that 550 R whole-body irradiation was the optimal dose for lymphoma development in the present testing system, we wondered whether there was a time threshold for thymus grafting after host irradiation. Seven-week-old male mice were thymectomized and exposed to 550 R whole-body irradiation, and thereafter grafted with a newborn thymus under the kidney capsule at 9, 30, 60, 90 and 105 days after irradiation. The same leukaemogenic filtrate preparation used throughout these experiments was injected directly into a 7-day-old thymus graft. The control group, in which irradiation of the host was omitted, was designed to show the leukaemogenic effect of the filtrate in non-irradiated host mice.

TABLE III.—*Persistence of the Radiation Effect in the Host*

| Treatment of host | Leukaemia incidence | Average latent period (days) |
|---|---|---|
| Thx —1 day— 550 R —9 days— i.r. Th —7 days— CFF | 20/24=83% | 106 |
| Thx —1 day— 550 R —30 days— i.r. Th —7 days— CFF | 11/18=61% | 112 |
| Thx —1 day— 550 R —60 days— i.r. Th —7 days— CFF | 7/18=40% | 140 |
| Thx —1 day— 550 R —90 days— i.r. Th —7 days— CFF | 12/26=46% | 130 |
| Thx —1 day— 550 R —105 days— i.r. Th —7 days— CFF | 4/18=22% | 156 |
| Thx —30 days— i.r. Th —7 days— CFF | 2/17=11% | 145 |

Thx = Thymectomy.
i.r. Th = Intrarenal thymus graft.
CFF = Cell-free filtrate.

As seen in Table III, the 9 day interval for thymus grafting after irradiation yielded an 83 per cent lymphoma incidence at an average latent period of 106 days. Postponing the thymus grafting to 30 days after irradiation decreased the lymphoma incidence slightly to 61 per cent, whereas after an interval of 60–90 days, the incidence of lymphoma development was reduced to 40–46 per cent, as compared to 83 per cent in hosts grafted 9 days after exposure to radiation. Delaying the thymus grafting to 105 days after irradiation minimized the radiation effect—only 22 per cent of the inoculated mice developed lymphomas, an incidence similar to that obtained in non-irradiated control mice (11 per cent). No significant differences were noted in the average latent period of tumour development in the different test groups.

*Immunological impairment in the irradiated mice*

Determination of the type of defects induced in the immunologically impaired mice by thymectomy and radiation, was carried out by testing the following systems:

(1) Circulating antibody production to Shigella antigen.

Male and female mice, 2 months old, were divided into 3 groups, and given the following treatment:

A. Thymectomy followed by 550 R whole-body exposure.
B. Thymectomy alone.
C. Normal control mice.

These groups were immunized with Shigella antigen at intervals of 24 hours, 7, 14, 21 and 30 days after completion of the irradiation treatment; the sera were collected 7 days after Shigella inoculation, and tested for agglutinating antibody to Shigella.

TABLE IV.—*Antibody Production by Irradiated C57Bl Mice Inoculated with Shigella Antigen*

| | Agglutinin titre at different intervals after irradiation: | | | | | | | |
|---|---|---|---|---|---|---|---|---|
| | 8 days | | 14 days | | 21 days | | 30 days | |
| Treatment of mice | $\log_2$ of titre (mean) | S.E. | $\log_2$ of titre (mean) | S.E. | $\log_2$ of titre (mean) | S.E. | $\log_2$ of titre (mean) | S.E. |
| A. Thx + 550 R W.B. | 3·5* | 0·5 | 4·4 | 0·45 | 7·3 | 0·61 | 12 | 0·41 |
| B. Thx | 10·5 | 0·15 | 11·2 | 0·32 | 10·1 | 0·25 | 11 | 0·18 |
| C. Normal | 11·6 | 0·27 | — | — | — | — | 12·4 | 0·26 |

S.E. = Mean standard error.
W.B. = Whole-body irradiation.
* 15 mice were used in each test group.

The results are summarized in Table IV. The immunological impairment induced by 550 R whole-body irradiation was transient. Minimal antibody production was found in these mice 8 days after irradiation, with a mean $\log_2$ titre of 3·5, as compared to 10·5 in the thymectomized non-irradiated mice, and 11·6 in normal controls. The low titres persisted for about 8 days after irradiation; thereafter a gradual increase was noted, reaching full recovery at about 30 days after irradiation.

(2) Homograft reaction to allogeneic grafts of a C3H/Jax transplantable fibrosarcoma.

Female mice, 5–7 weeks old, were thymectomized and within a week exposed to 550 R, 300 R or 75 R × 2 (at weekly intervals) whole-body irradiation. The allogeneic cell suspension of a C3H/Jax transplantable fibrosarcoma was injected into the host mice at different intervals after irradiation, as well as into thymectomized non-irradiated mice. The effect of irradiation on postponing or abolishing the spontaneous reactivation of the homograft reaction against the genetically-foreign C3H tumour is described in Table V. All the tumour grafts in the thymectomized non-irradiated control mice were rejected, and none showed palpable growth before their rejection. Thymectomized mice exposed to 550 R whole-body irradiation displayed a state of suppression of the immune mechanism even at 90 days after irradiation: 12 out of 14 mice exhibited tumour growth, 7 of which

TABLE V.—*Immunological Reactivity of Thymectomized Irradiated C57Bl Mice to Allogeneic Grafts of a C3H/Jax Fibrosarcoma*

| Treatment of host | No. of takes/ No. of mice | Lethal takes/ No. of mice | Mean time of regression (days) | Mean time of death (days) |
|---|---|---|---|---|
| Thx —7 days— 550 R —9 days— C3HT | 17/17 | 17/17 | — | 36 |
| Thx ——— C3HT | 0/17 | — | — | — |
| Thx —7 days— 550 R —22 days— C3HT | 16/17 | 16/17 | — | 51 |
| Thx ——— C3HT | 0/17 | — | — | — |
| Thx —7 days— 550 R —90 days— C3HT | 12/14 | 7/14 | 17 | 47 |
| Thx ——— C3HT | 0/15 | — | — | — |
| Thx —7 days— 300 R —1 day— C3HT | 8/10 | 2/10 | 16 | 32 |
| Thx —7 days— 300 R —7 days— C3HT | 6/10 | 0/10 | 21 | — |
| Thx —7 days— 300 R —30 days— C3HT | 7/9 | 2/10 | 15 | 61 |
| Thx —9 days— 300 R —45 days— C3HT | 0/10 | — | — | — |
| Thx —7 days— 75 R × 2 —2 days— C3HT | 0/20 | — | — | — |
| Thx —7 days— 75 R × 2 —45 days— C3HT | 0/15 | — | — | — |

Thx = Thymectomy.
C3HT = C3H tumour graft (i.m.).

were lethal. At 9 and 22 day intervals in the 550 R exposure groups, all the mice developed lethal tumour growth. Exposure of thymectomized mice to 300 R irradiation was less effective than the 550 R dose—no tumour growth was noted at a 45-day interval, while shorter intervals resulted in 60–80 per cent of the grafted mice developing temporary tumour growth, which then regressed within 15–21 days after first becoming palpable. The low dose of 75 R × 2 did not exert any effect on the immune mechanism of the irradiated mice.

*Irradiation of the host after inoculation of the leukaemogenic agent into the thymus graft*

We wished to test whether the irradiation of the host would also be effective if applied after the inoculation of the leukaemogenic agent into the thymus graft. Male C57Bl mice, 7–8 weeks old, were thymectomized and 2 days later implanted with a newborn thymus under the kidney capsule. When the thymus graft was 7 days old the leukaemogenic filtrate was injected directly into these grafts. One group of these mice was left without further treatment as a control group; 4 other groups were exposed to 550 R whole-body irradiation at 8, 30, 60 or 90 days after inoculation of the leukaemogenic agent. The effective potency of the leukaemogenic filtrate used in this experiment was tested in the usual way: thymectomized mice were exposed to 550 R whole-body irradiation, thereafter a newborn thymus was grafted under the kidney capsule, and the leukaemogenic agent was injected into a 7-day-old graft. The possible leukaemogenic effect of the radiation exposure on the thymus graft was also tested, by exposing thymectomized mice carrying a 15- or 37-day-old thymus graft in the kidney to 550 R whole-body irradiation.

The results are reported in Table VI. A similar high incidence of 95–100 per cent leukaemia development, having a similar latent period of 100–120 days, was

TABLE VI.—*The Effect of Host Irradiation After Inoculation of Leukaemogenic Filtrate into Thymus Graft*

| Treatment of host | Leukaemia incidence | Average latent period (days) |
|---|---|---|
| A. Thx —2 days— ir. Th —7 days— CFF | 3/15=20% | 105 |
| B. Thx —7 days— 550 R W.B. —1 day— i.r. Th —7 days— CFF | 19/20=95% | 100 |
| C. Thx —2 days— i.r. Th —7 days— CFF —8 days— 550 R W.B. | 16/16=100% | 120 |
| D. Thx —2 days— i.r. Th —15 days— 550 R W.B. | 4/24=16% | 184 |
| E. Thx —2 days— i.r. Th —7 days— CFF —30 days— 550 R W.B. | 9/15=60% | 223 |
| F. Thx —2 days— i.r. Th —37 days— 550 R W.B. | 0/13 | — |
| G. Thx —2 days— i.r. Th —7 days— CFF —60 days— 550 R W.B. | 2/15=13% | 265 |
| H. Thx —2 days— i.r. Th —7 days— CFF —90 days— 550 R W.B. | 3/15=20% | 140 |

Thx = Thymectomy.
i.r.Th = Intrarenal thymus graft.
CFF = Cell-free leukaemogenic filtrate.
W.B. = Whole-body exposure.

observed whether irradiation was performed shortly before or after inoculation of the leukaemogenic filtrate (groups B and C), but when the irradiation was omitted (group A), only 20 per cent of the inoculated mice developed lymphomas, at a mean latent period of 105 days. The leukaemogenic effect of 550 R exposure to a non-inoculated thymus graft was very mild—only 16 per cent of these mice developed tumours (group D). Postponing the irradiation to 30 days after inoculation of the filtrate produced a decrease in the leukaemia incidence to 60 per cent, and lengthened the average latent period to 223 days (group E); the matching irradiated control group was negative (group F). Delaying the exposure to irradiation to 60 or 90 days after inoculation of the leukaemogenic filtrate nullified its effectiveness (groups G and H), the results being similar to those obtained in the non-irradiated control group (group A).

### DISCUSSION

Effective means for lymphoma induction by the leukaemogenic filtrable agent isolated from irradiated C57Bl mice is its direct inoculation into the thymus of neonatal C57Bl mice, or into the thymus graft of the thymectomized irradiated adult host (Haran-Ghera, Lieberman and Kaplan, 1966). This testing system is not effective in adult hosts when irradiation of the host is omitted, even though the agent is inoculated into a thymus graft in a proliferative state with many large and medium lymphocytes, which are considered by Kaplan (1961) to be the susceptible thymocytes for neoplastic transformation. Irradiation could therefore be proposed as an important factor in neoplastic transformation and/or proliferation, perhaps by causing transient depression of the immune response of the host. This assumption has been tested experimentally in the present studies, by observing the effect of immune reactivation of the host versus immune impairment on lymphoma induction, and its relation to X-ray dosage. Immune restoration of the irradiated host submitted to 550 R irradiation with shielding of bone marrow or spleen decreased the lymphoma incidence when compared to whole-body exposure (27–36 per cent versus 88 per cent) (Table I). The injection of spleen and lymph node cells shortly after irradiation, as another means for immune reactivation of the host, was less effective in the present studies (56 per cent versus 88 per cent), perhaps because the amount of cells injected was not sufficient.

It was also shown that the increase in lymphoma development was related to dosage of X-ray exposure to the host (Table II): 150 R and smaller doses were ineffective; 250–300 R were increasingly effective, and 550 R was maximally effective, results that coincide with those of Taliaferro, Taliaferro and Jaroslow (1964) on the relation of X-ray dosage and immune impairment. There seems to be a threshold dose of X-rays, similar to the minimal X-ray dose that can produce transient immune depression, which is essential for neoplastic proliferation in the present testing system. The results have indicated a parallelism between the X-ray dosage that causes different degrees of immune impairment and lymphoma incidence in the sensitive test system for lymphoma development used in the present studies.

It is interesting to note that fractionated irradiation (170 R $\times$ 4) did not display any preference to a single high dose exposure to the host, though fractionated irradiation is a more favourable method for radiation leukaemogenesis (Kaplan and Brown, 1952), suggesting that this procedure may be related to one of the other effects involved in radiation leukaemogenesis, such as thymus and bone marrow injury.

In the present studies, 550 R whole-body irradiation was found to be the optimal dose for lymphoma development in the testing system used. It was found that this radiation dose has a prolonged effect on the host—even 90 days after exposure to 550 R the thymectomized irradiated host was still susceptible to lymphoma development when the leukaemogenic filtrate was injected into a several-day old thymus grafted under the kidney capsule of this pretreated host (Table III). On the assumption that the transient depression of the immunological response induced by irradiation was the contributor to neoplastic proliferation, we wondered whether this immune impairment induced by exposure of thymectomized mice to 550 R whole-body irradiation could persist for so long a period of about 3 months. As the immunological impairment includes defects in circulating antibody formation, homograft rejection and the delayed type of hypersensitivity, and need not be a generalized one, we tested these irradiated mice for defects in their capacity to produce antibodies to the Shigella antigen, and also the homograft rejection ability by these thymectomized irradiated mice of an allogeneic tumour graft. The present experiments (Table IV) showed that 550 R whole-body irradiation of thymectomized mice caused a marked decrease in antibody production to the Shigella antigen (evaluated by the amount of agglutinin formation), but the low titres persisted for about 8 days after irradiation; thereafter a gradual increase was noted, reaching full recovery to normal values at about 30 days after irradiation. These results did not coincide with our previous findings that the effect of irradiation, contributing to neoplastic proliferation, persisted for a much longer period than 30 days, and led us to assume that another type of defect in the immune mechanism may be involved. Feldman and Globerson (1964) have shown that thymectomy of adult mice followed by whole-body irradiation with sublethal doses caused defects in their immune response—these mice could not reject allogeneic tumour tissue grafted into them 7 or 40 days after irradiation. We have applied this same testing system of an allogeneic tumour graft to a longer interval of 90 days after irradiation (the effective time interval in the present testing system), and to different radiation doses that were found to act differently on lymphoma development. Indeed, the tests used in this study for homograft reaction to allogeneic grafts of a C3H/Jax transplantable sarcoma have shown that thymectomized mice exposed to 550 R whole-body irradiation exhibited a state of suppression of the immune mechanism. The allogeneic tumour grafted into C57Bl mice 9 or 22 days after irradiation grew progressively and killed the host mice, and even 90 days after the irradiation these thymectomized irradiated (550 R) mice could still delay or abolish the spontaneous homograft reaction against genetically foreign tumours. The low dose of 75 R $\times$ 2 did not have any effect on the host's immune mechanism, expressed by homograft rejection. The exposure to 300 R was effective, though less so than the 550 R irradiation. An allogeneic tumour grafted into thymectomized C57Bl mice 1–30 days after their exposure to 300 R, showed a 60–80 per cent tumour take, though many of these tumours regressed within 15–20 days after first becoming palpable, whereas 45 days after exposure to 300 R all these allogeneic tumour grafts were rejected, as in non-irradiated normal C57Bl mice. These results are in accord with our findings (Haran-Ghera, 1967, unpublished observations) that thymectomized mice exposed to 300 R develop a high incidence of lymphomas if the grafting and filtrate inoculation are carried out within 1–30 days after irradiation. But postponing the testing procedure to 45 days after irradiation nullifies the radiation effect, and the lymphoma induction rate in these mice is similar to that in non-irradiated control mice.

The results suggest that cellular immunity impairment of the homograft reaction type, which is induced by irradiation of the host, and persists for several months, may be involved in the neoplastic proliferation. Law, Ting and Leckband (1966) have indicated that the most plausible explanation for polyoma virus oncogenesis is related to the immunological capacity of the host, and that cellular immunity of the homograft type produced against a virus-specific "tumour" antigen, which is present in the neoplastic cells, is involved. The present results may suggest a similar pathway for the leukaemogenic filtrable agent isolated from irradiated tissues of C57Bl mice.

Following the results obtained on the important contribution of irradiation of the host (before inoculation of the filtrate into the several-day old thymus graft) in neoplastic proliferation, we wondered whether irradiation of the host would also be effective if applied after the inoculation of the filtrate into the intrarenal thymus graft, and if this is so, for how long could the irradiation of the host be effectively delayed after virus inoculation. The present experiments indicated that irradiation of the host was equally effective whether applied shortly before or after thymus grafting and inoculation of the agent; but delaying the irradiation to 30 days after inoculation of the filtrate caused a decrease in leukaemia incidence, and lengthened the mean latent period of tumour development (Table VI). When irradiation was applied to the host mice 60 or 90 days after injection of the leukaemogenic filtrate, it had no effect on lymphoma induction—the tumour incidence was similar to that in non-irradiated inoculated control mice. Whether these variable results are due to loss of viability and activity by the leukaemogenic agent, or to some immune mechanism, is now under investigation.

In conclusion, the present results propose that transient depression of immunologic responsiveness of the host, caused by its exposure to X-rays, may be an important factor in neoplastic transformation and/or proliferation induced by the leukaemogenic transmissible agent isolated from irradiated C57Bl mouse tissues.

SUMMARY

Experimental evidence has been provided in support of the assumption that radiation may contribute to X-ray leukaemogenesis in C57Bl mice, by causing transient depression of the immune mechanism, in addition to its known effects on thymus and bone marrow injury and the " release " of the leukaemogenic agent.

The use of the sensitive testing system of injecting the leukaemogenic filtrate into a several-day-old thymus implant under the kidney capsule of thymectomized irradiated mice (550 R whole-body exposure) was essential in analysing this hypothesis. In this testing system, if irradiation was omitted, there was a very marked reduction in the lymphoma incidence, even though the leukaemogenic agent was inoculated into the test mice in abundance. Immune reactivation of the irradiated hosts by bone marrow or spleen shielding, or by injection of spleen and lymph node cells shortly after irradiation, caused a significant decrease in the incidence of lymphoma induction. The rate of lymphoma development was related to dosage of X-ray exposure to the host. The effective minimal X-ray dose for tumour development was 250–300 R, while 550 R whole-body irradiation was maximally effective, results that coincide with the relation of X-ray dosage to induction of immune impairment. Studies to determine the type of the immune defect caused by irradiation suggest that cellular immunity impairment of the homograft reaction type may be involved in the neoplastic proliferation.

It has been shown that irradiation of the host was equally effective whether applied shortly before or after agent inoculation, but delaying the radiation beyond 30 days after the leukaemogenic filtrate inoculation caused a progressive decrease in the leukaemia incidence.

The author is indebted to Mrs. A. Peled for testing the production of circulating antibodies in the irradiated mice, and to Mr. S. Yecheskel for his competent technical assistance.

REFERENCES

Axelrad, A. A. and Van der Gaag, H. C.—(1962) *J. natn. Cancer Inst.*, **28**, 1065.
Feldman, M. and Globerson, A.—(1964) *Ann. N.Y. Acad. Sci.*, **120**, 182.
Gross, L.—(1958) *Acta haemat.*, **19**, 353.
Haran-Ghera, N.—(1966) *Int. J. Cancer*, **1**, 81.
Haran-Ghera, N., Lieberman, M. and Kaplan, H. S.—(1966) *Cancer Res.*, **26**, 438.
Haran-Ghera, N. and Peled, A.—(1967) *Brit. J. Cancer*, **21**, 730.
Jenkins, V. K. and Upton, A. C.—(1963) *Cancer Res.*, **23**, 1748.
Kaplan, H. S.—(1957) in 'The Leukemias, Etiology, Pathology and Treatment'. New York (Academic Press), p. 163.—(1961) *Cancer Res.*, **21**, 981.—(1964) *Natn. Cancer Inst. Monogr.*, **14**, 207.
Kaplan, H. S. and Brown, M. B.—(1952) *J. natn. Cancer Inst.*, **13**, 185.
Kaplan, H. S., Brown, M. B. and Paull, J.—(1953) *J. natn. Cancer Inst.*, **14**, 303.
Law, L. W., Ting, R. C. and Leckband, E.—(1966) in *Ciba Fdn Symp.* 'The Thymus'. London (J. & A. Churchill, Ltd.), p. 214.
Lieberman, M. and Kaplan, H. S.—(1959) *Science, N.Y.*, **130**, 387.
Lorenz, E., Congdon, C. C. and Uphoff, D.—(1953) *J. natn. Cancer Inst.*, **14**, 291.
Taliaferro, W. H., Taliaferro, L. G. and Jaroslow, B. M.—(1964) in 'Radiation and Immune Mechanism'. New York and London (Academic Press) p. 31.